# IL-17E synergizes with EGF and confers *in vitro* resistance to EGFR-targeted therapies in TNBC cells

**DOI:** 10.18632/oncotarget.10804

**Published:** 2016-07-23

**Authors:** Yacine Merrouche, Joseph Fabre, Herve Cure, Christian Garbar, Camille Fuselier, Jeremy Bastid, Frank Antonicelli, Reem Al-Daccak, Armand Bensussan, Jerome Giustiniani

**Affiliations:** ^1^ Institut Jean Godinot, Unicancer, F-51726 Reims, France; ^2^ Université Reims-Champagne-Ardenne, DERM-I-C, EA7319, 51095 Reims, France; ^3^ CHU-Grenoble Alpes, CS 10217, 38043 La Tronche, France; ^4^ Institut National de la Santé et de la Recherche Médicale (INSERM) U823, Centre de Recherche (CRI), Institut Albert Bonniot, 38043 La Tronche, France; ^5^ OREGA Biotech, F-69130 Ecully, France; ^6^ Institut National de la Santé et de la Recherche Médicale (INSERM) UMR-S 976, Hôpital Saint Louis, 75010 Paris, France; ^7^ Université Paris Diderot, Sorbonne Paris Cité, Laboratoire Immunologie Dermatologie and Oncologie, UMR-S 976, F-75475, Paris, France

**Keywords:** IL-17E, breast cancer, EGFR, resistance, TNBC

## Abstract

Estrogen receptor-, progesterone receptor- and HER2-negative breast cancers, also known as triple-negative breast cancers (TNBCs), have poor prognoses and are refractory to current therapeutic agents, including epidermal growth factor receptor (EGFR) inhibitors. Resistance to anti-EGFR therapeutic agents is often associated with sustained kinase phosphorylation, which promotes EGFR activation and translocation to the nucleus and prevents these agents from acting on their targets. The mechanisms underlying this resistance have not been fully elucidated. In addition, the IL-17E receptor is overexpressed in TNBC tumors and is associated with a poor prognosis. We have previously reported that IL-17E promotes TNBC resistance to anti-mitotic therapies. Here, we investigated whether IL-17E promotes TNBC resistance to anti-EGFR therapeutic agents by exploring the link between the IL-17E/IL-17E receptor axis and EGF signaling. We found that IL-17E, similarly to EGF, activates the EGFR in TNBC cells that are resistant to EGFR inhibitors. It also activates the PYK-2, Src and STAT3 kinases, which are essential for EGFR activation and nuclear translocation. IL-17E binds its specific receptor, IL-17RA/IL17RB, on these TNBC cells and synergizes with the EGF signaling pathway, thereby inducing Src-dependent EGFR transactivation and pSTAT3 and pEGFR translocation to the nucleus. Collectively, our data indicate that the IL-17E/IL-17E receptor axis may underlie TNBC resistance to EGFR inhibitors and suggest that inhibiting IL-17E or its receptor in combination with EGFR inhibitor administration may improve TNBC management.

## INTRODUCTION

Triple-negative breast cancer (TNBC) is a heterogeneous disease comprising several biologically distinct subtypes, each of which is associated with a distinct gene ontology and drug sensitivity [1, 2]. Currently, TNBC is managed mainly with chemotherapy, because no targeted therapies have been approved for the treatment of this disease. Nevertheless, nearly 50% of TNBC tumors overexpress the epidermal growth factor receptor (EGFR) [3, 4], thus suggesting that the EGFR may serve as a molecular marker of these tumors and that the EGFR pathway may have promise as a therapeutic target in TNBC management [4]. Agents such as monoclonal antibodies that bind the extracellular ligand-binding domain of the EGFR (e.g., Cetuximab) or small molecules that inhibit the intracellular tyrosine kinase domain of the EGFR (e.g., Gefitinib/Iressa) have shown only limited effectiveness against TNBC because resistance frequently and rapidly develops; thus, metastatic TNBC often has a poor prognosis. Only 10–20% of TNBC patients show marked clinical improvement in response to therapy [5, 6]. Thus, improving the efficacy of anti-EGFR therapy in TNBC is needed.

EGFR is a plasma membrane-bound receptor tyrosine kinase that initiates growth and survival signals but can also localize to and function from the nucleus [7–9]. Thus, TNBCs rely on two distinct types of EGFR signaling to sustain their oncogenic phenotype” classical membrane-bound EGFR signaling and nuclear EGFR (nEGFR) signaling. The actions of both types of signaling promote TNBC resistance to anti-EGFR therapeutic agents [7, 10]. One mechanism underlying these actions is crosstalk between the EGFR and other signaling proteins, such as c-met and c-Src [11]. The paracrine pathways that are active within the TNBC microenvironment and facilitate the crosstalk promoting EGFR resistance have not been fully elucidated.

IL-17E is a member of the pro-inflammatory IL-17 cytokine family, which binds the IL-17RA/IL17RB complex. It is highly expressed in certain organs, including the testis and pancreas, and is not highly expressed in other organs, such as normal breast [12, 13]. Normal mammary epithelial cells (MECs) transiently produce IL-17E during mammary gland development, and IL-17E acts in conjunction with other MEC-secreted factors in preventing malignant cell growth [14]. Consistently with these findings, our recent report has demonstrated that IL-17E is scarcely detectable in normal adult breast tissues but is expressed in some tumor tissues, including TNBC tissues, as a component of their microenvironment [15]. IL-17E causes apoptosis in breast cancer cells expressing its receptor [14], and its secretion by tumor-associated fibroblasts suppresses the growth of human mammary tumor MDA-MD-231 cells serving as a metastasis control checkpoint [16]. Paradoxically, the IL-17E receptor subunits IL17-RA and RB are overexpressed in TNBC tumors [15], and IL17-RB expression is associated with a poor prognosis [17]. Furthermore, we have shown that, similarly to IL-17A, IL-17E does not induce cell death by binding to its receptor on TNBC cells but instead activates oncogenic pathways, such as c-raf and p70S6 pathways, thus resulting in Docetaxel resistance [15]. The signaling cascades downstream of the IL-17E receptor have never been explored with respect to TNBC resistance to anti-EGFR therapeutic agents.

This study builds on our previous report [15] and explores the crosstalk between IL-17E and EGF signaling in the context of anti-EGFR-resistant TNBC tumors and the eventual contribution of this crosstalk to therapy resistance. We found that IL-17E and EGF trigger interconnected molecular signaling pathways in TNBC cells through their specific receptors, thus suggesting that EGFR/IL-17RB crosstalk promotes TNBC resistance to anti-EGFR therapeutic agents. Hence, our findings provide the first evidence of the potential of the IL-17E/IL-17RB axis as a therapeutic target in the management of TNBC tumors and eventually other EGFR and IL-17RA/RB co-expressing tumors.

## RESULTS

### IL-17E phosphorylates the EGFR in Iressa-resistant TNBC cell lines and potentiates their resistance

**Figure 1:**
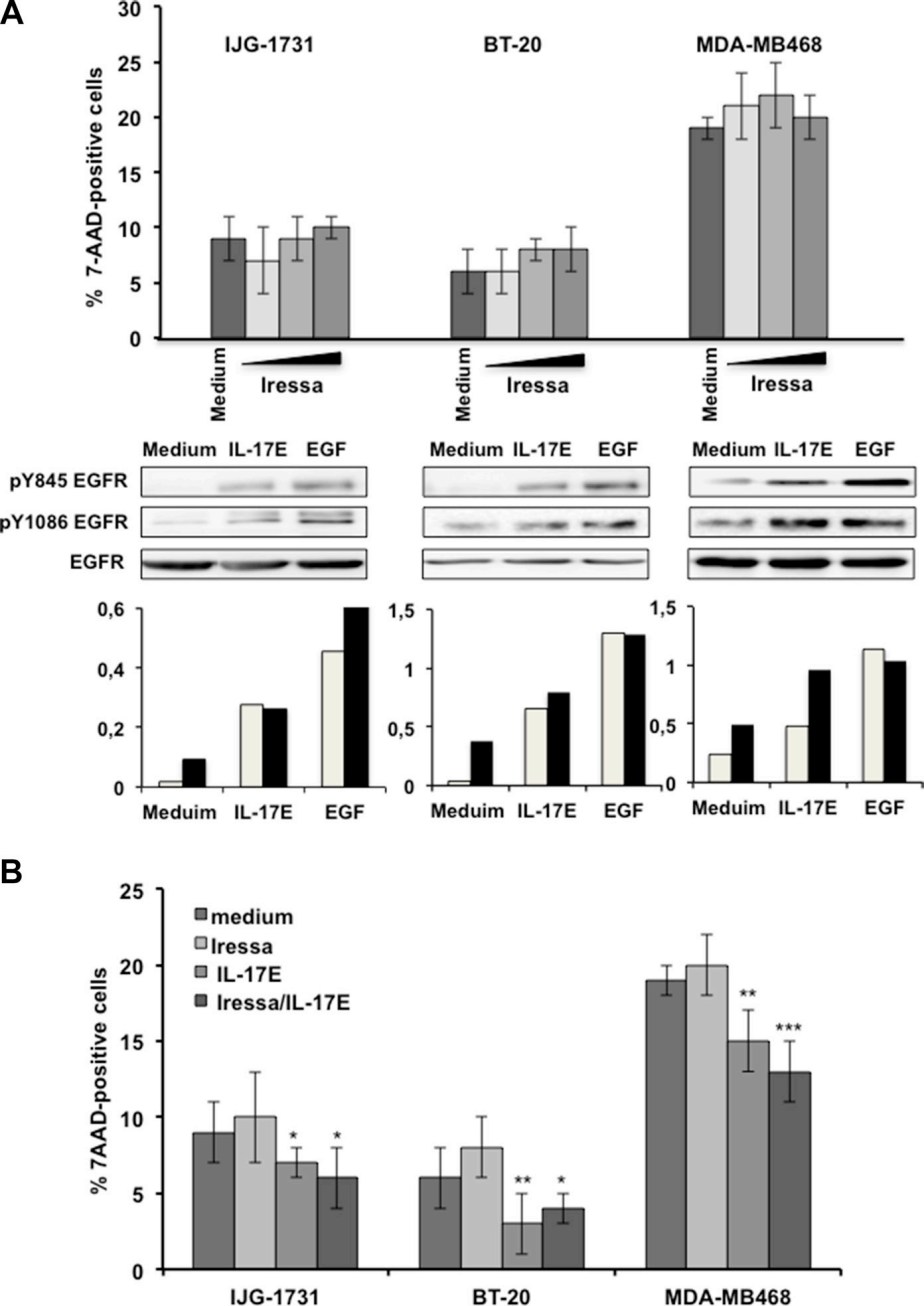
IL-17E phosphorylates the EGFR in Iressa-resistant TNBC cell lines (**A**) In the upper panel, IJG-1731, BT20, and MDA-MB468 TNBC cells were cultured alone or in the presence of increasing concentrations of the EGFR inhibitor Iressa (0.25 (■), 0.5 (■), and 1 (■) μM) for 48 hours, and cell death was evaluated by determining the percentage of 7AAD-positive cells and by flow cytometry analysis. The results are presented as the mean ± SD of three independent experiments performed in triplicate. In the middle panel, TNBC cells were cultured alone or in the presence of IL-17E (10 ng/ml) or EGF (10 ng/ml), and the phosphorylation of EGFR at residues Y845 and Y1086 was assessed by western blotting. Membranes were re-blotted with anti-EGF as a loading control. Data are representative of 3 independent experiments. In the lower panel, densitometric quantification of EGFR phosphorylation, as shown in the representative blots, is expressed as the ratios of pY845 EGFR to EGFR (■) and pY1086 EGFR to EGFR (■). (**B**) IJG-1731, BT20, and MDA-MB468 TNBC cells were cultured alone or in the presence of Iressa (1 μM), IL-17E (10 ng/ml), or a combination of both for 48 hours, and the percentage of 7AAD-positive cells was determined by flow cytometry. The results are presented as the mean ± SD of three independent experiments performed in triplicate. Student *t*-test was used (*P < 0.05; **P < 0.01; ***P < 0.001) compared with medium alone.

TNBC *ex-vivo*-derived IJG-1731 cells and BT20 and MDA-MB468 cells are established TNBC tumors models that exhibit pronounced resistance to a specific inhibitor of EGFR phosphorylation, Iressa, even when exposed to high concentrations (1 μM) of this inhibitor for 48 hours (Figure 1A upper panel). IJG-1731, BT-20 and MDA-MB468 cells exhibit different levels of EGFR expression and distinct Y845 EGFR and Y1086 EGFR phosphorylation patterns (Y845 EGFR is a substrate for Src kinase, and Y1086 EGFR is directly phosphorylated by EGFR) [18, 19] after treatment with EGF (10 ng/ml), thus reflecting the heterogeneity of TNBC tumors (Figure 1A lower panel). Similarly to EGF treatment, IL-17E treatment (10 ng/ml) induced the phosphorylation of both Y845 EGFR and Y1086 EGFR (Figure 1A lower panel). The intensity of IL-17E-induced EGFR phosphorylation was comparable to that of EGF-induced EGFR phosphorylation and was consistent with the basal levels of EGFR expression in the cell lines. Similarly to Iressa, IL-17E (10 ng/ml) did not induce cell death, either alone or in combination with Iressa (1 μM), in any of the cell lines; instead, it potentiated resistance to EGFR inhibitors (Figure 1B). Together, these results suggest that EGFR and IL-17E signaling may interact and together sustain TNBC resistance to EGFR inhibitors.

### IL-17E promotes EGFR phosphorylation in TNBC cell lines

**Figure 2:**
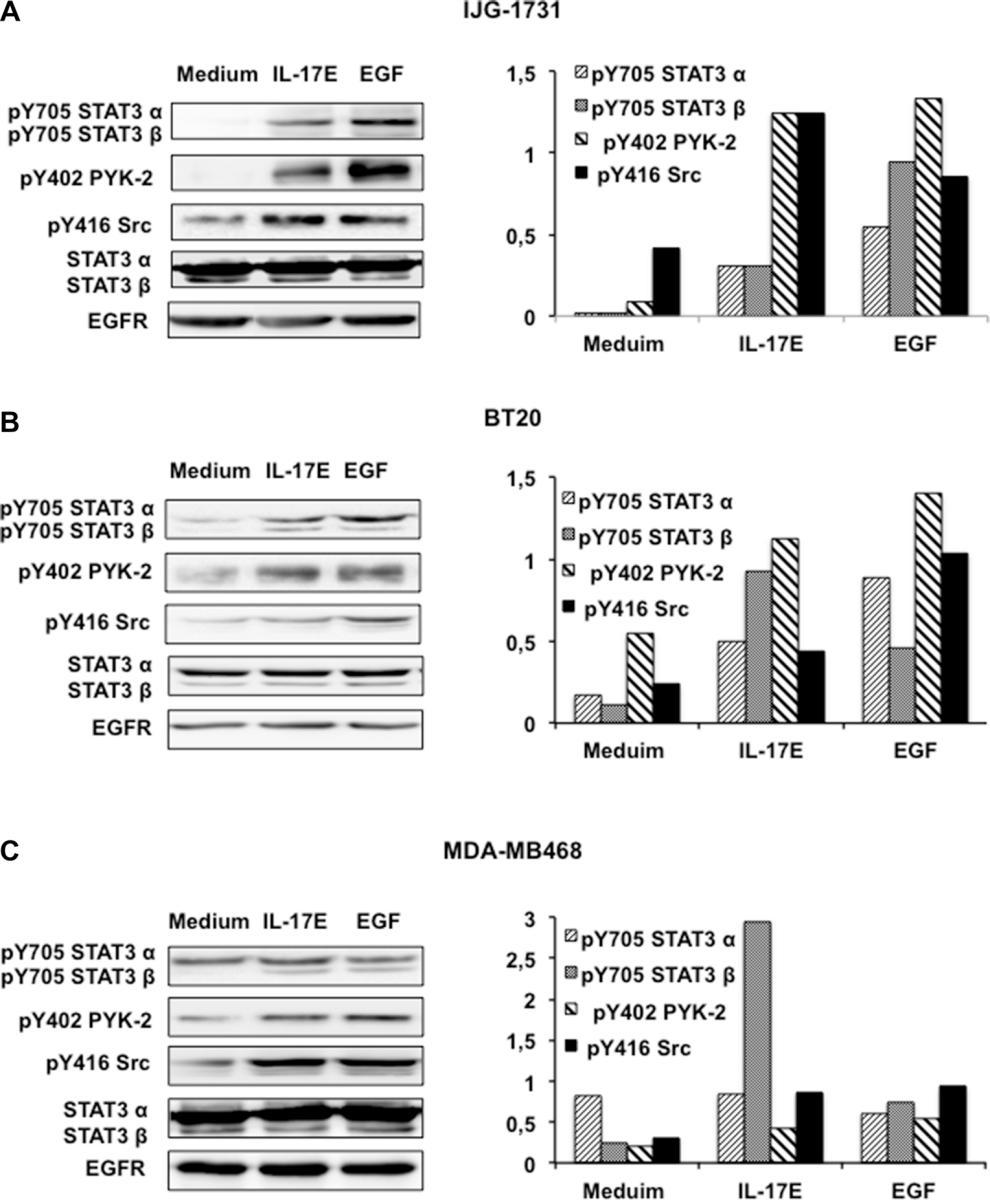
IL-17E phosphorylates the kinases essential for EGFR activation IJG-1731 (**A**), BT20 (**B**), and MDA-MB468 (**C**) cells were cultured alone or in the presence of IL-17E (10 ng/ml) or EGF (10 ng/ml), and then STAT3 phosphorylation at Y705, PYK-2 phosphorylation at Y402 and Src phosphorylation at Y416 were assessed by western blotting (left panel). Membranes were re-blotted with anti-EGF or anti-STAT3α/β antibodies, which served as loading controls. Data are representative of 3 independent experiments. In the right panel, densitometric quantification of STAT3a/b, PYK-2 and Src phosphorylation, as shown in the representative blots, is expressed as the ratios of pY705 STAT3a and b to their respective un-phosphorylated forms, pY402 PYK-2, pY416 Src and EGFR, as indicated.

Previous studies have shown that STAT3, PYK-2, and Src kinase phosphorylation is essential for EGFR phosphorylation [20]. Consequently, we examined the phosphorylation statuses of these essential kinases in the three cell lines treated with IL-17E. Similarly to EGF, IL-17E induced considerable STAT3-α and β phosphorylation at Y705 in IJG-1731 and BT20 cells (Figure 2A and 2B). The phosphorylation levels of both STAT3-α and β were in accordance with the phosphorylation levels of Y1086 and Y845 EGFR in these cell lines (Figure 1A). IL-17E-induced STAT3-α and β phosphorylation was less evident in MDA-MB468 cells (Figure 2C), probably because of elevated STAT3-α phosphorylation, but was consistent with IL-17E-induced EGFR phosphorylation levels (Figure 1A). Treatment with IL-17E also induced PYK2 and Src kinase phosphorylation at residues Y402 and Y416, respectively, in the three cell lines at levels comparable to those induced by EGF (Figure 2).

Thus, IL-17E and EGF similarly phosphorylate the essential kinases implicated in EGFR phosphorylation; hence, IL-17E may contribute to TNBC resistance to EGFR inhibitors.

### IL-17E signaling interacts with EGF signaling

**Figure 3:**
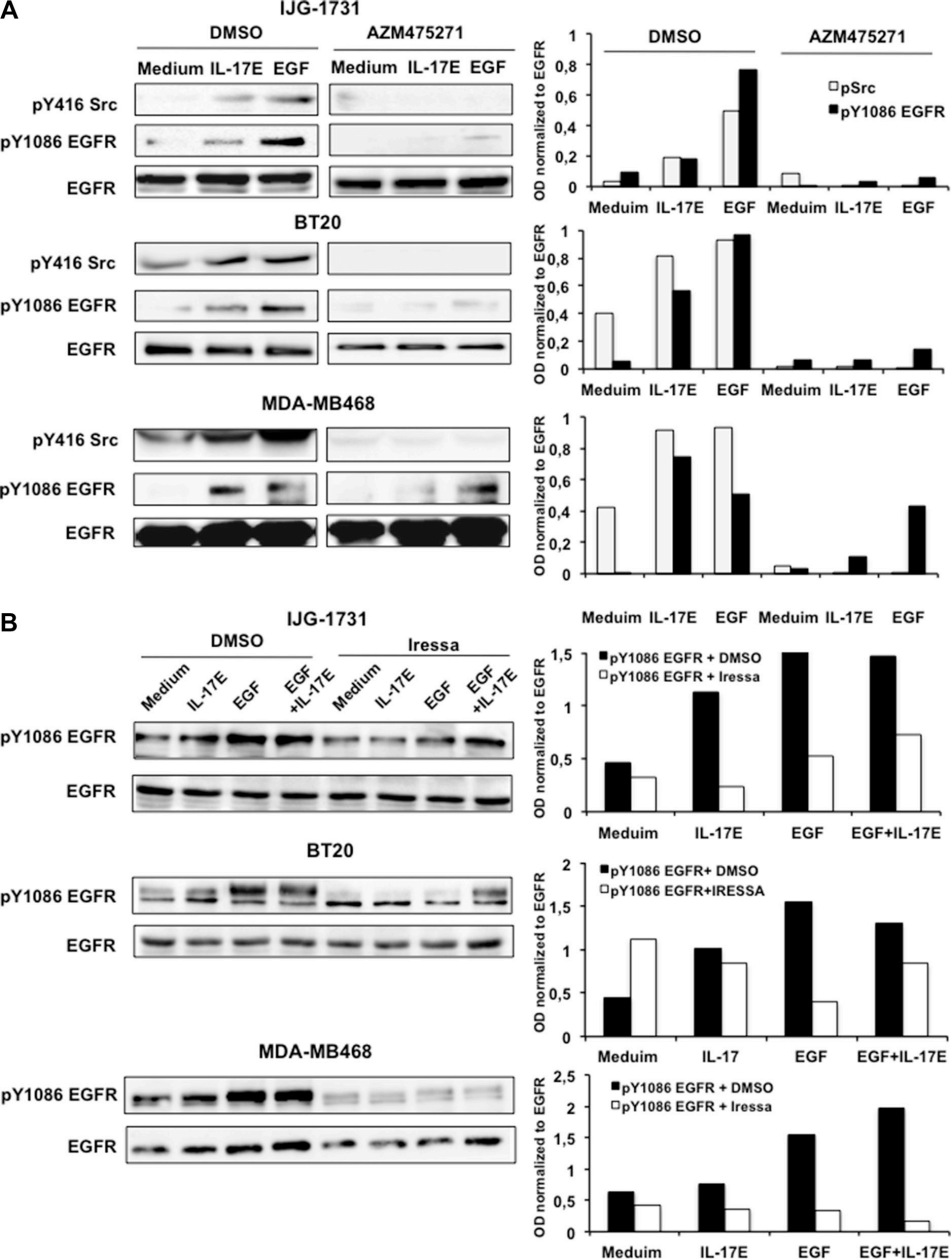
IL-17E-induced EGFR phosphorylation is dependent on Src and EGFR kinase activity IJG-1731, BT20, and MDA-MB468 cells were treated with the Src specific inhibitor AZM475271 (10 μM) (**A**), Iressa (0.25 μM) (**B**), or control DMSO and then stimulated with IL-17E (10 ng/ml), EGF (10 ng/ml) or with medium alone. EGFR and Src phosphorylation was then assessed by western blotting (left panel). Loading controls were determined by re-blotting the membranes with an anti-EGFR antibody. Data are representative of at least 2 independent experiments. In the right panel, densitometric quantification of Y416 Src and Y1086 EGFR, as shown in the representative blots, is expressed as the ratios of pY416 Src to EGFR and pY1086 EGFR to EGFR, as indicated.

To substantiate the contributions of IL-17E to TNBC resistance to EGFR inhibitors, we examined the interactions between IL-17E- and EGF-induced signaling. Sustained EGFR activity requires both Src and EGFR activation [16]. Therefore, we first determined the involvement of Src kinase in IL-17E-induced EGFR phosphorylation. TNBC tumor cell lines were pre-treated with the Src kinase-specific inhibitor AZM475271 and then stimulated with either IL-17E or EGF. Treatment with AZM475271 inhibited IL-17E- and EGF-induced Src phosphorylation but also abolished Y1086 EGFR phosphorylation in IJG-1731 and BT20 cells and, to a lesser extent, in MDA-MB468 cells (Figure 3A). Thus, similarly to EGF-induced EGFR phosphorylation, IL-17E-induced EGFR phosphorylation is also Src-dependent. This result suggests that IL-17E and EGF can transactivate the EGFR in TNBC tumors.

We then examined whether IL-17E-induced EGFR phosphorylation requires EGFR activity. TNBC cell lines were treated with Iressa, the specific inhibitor of EGFR phosphorylation, and then stimulated with IL-17E, EGF or a combination of both, and EGFR phosphorylation status was analyzed by western blotting. Treatment with Iressa elicited similar decreases in IL-17E- and EGF-induced Y1086-EGFR phosphorylation in the three cell lines (Figure 3B) and markedly decreased EGFR tyrosine phosphorylation induced by the combination of IL-17E and EGF in BT20 and MDA-MB468 cells and, to a lesser extent, in IJG-1731 cells (Figure 3B). Altogether, these data indicate that, similarly to EGF-induced phosphorylation, IL-17E-induced EGFR phosphorylation requires both Src and EGFR kinase activity; thus, EGF and IL-17E are interconnected and may synergistically activate and sustain EGFR phosphorylation in TNBC.

### IL-17E synergizes with EGF through its specific receptor IL17RA/IL17RB

**Figure 4:**
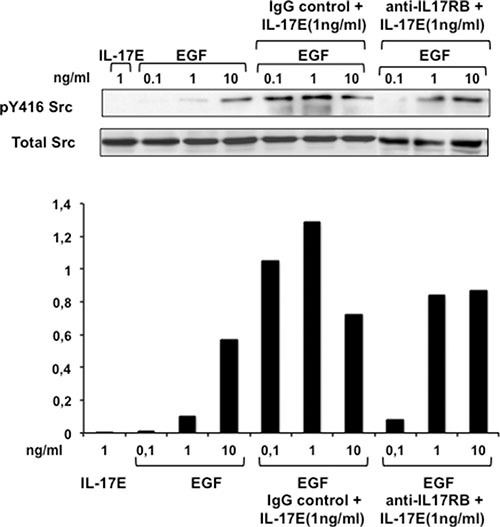
IL-17E synergizes with EGF in phosphorylating Src kinase MDA-MB468 cells were left untreated or treated with anti-IL-17RB mAb (10 μg/ml) or its isotype IgG control and then stimulated with IL-17E (1 ng/ml), EGF (0.1–10 ng/ml), or a combination of IL-17E (1 ng/ml) and various concentrations of EGF (0.1–10 ng/ml). Src phosphorylation (p416Src) was then assessed by western blotting using specific anti-pSrc antibodies. Re-blotting with anti-Src antibody was performed to determine equal loading. Data are representative of 2 independent experiments. In the lower panel, densitometric quantification of Y416 Src, as shown in the representative blots, is expressed as the ratio of pY416 Src to total Src.

Src pathway activation is essential for optimal EGFR activity. Therefore, to explore the synergistic effects of IL-17E and EGF on EGFR activation, we examined the phosphorylation status of Src in TNBC cells stimulated with increasing concentrations of EGF in the presence or absence of IL-17E. Stimulation of MDA-MB468 cells with IL-17E or EGF at suboptimal concentrations of 1 ng/ml and 0.1–1 ng/ml, respectively, did not induce significant Src phosphorylation at Y416 (Figure 4). However, stimulation with 1 ng/ml IL-17E and suboptimal concentrations of EGF (0.1 and 1 ng/ml) induced a level of Y416Src phosphorylation similar to that induced by 10 mg/ml EGF alone (Figure 4). These results indicate that IL-17E and EGF synergistically activate Src kinase. The presence of the anti-IL-17E receptor (IL-17RA/RB)-blocking antibody, but not its isotype control, substantially decreased Src phosphorylation at Y416 by 0.1 and 1 ng/ml EGF in the presence of IL-17E (Figure 4). Thus, the synergistic effects of IL-17E are mediated by its recruitment to its specific receptor, IL-17RA/RB.

### IL-17E facilitates pEGFR and pSTAT3 translocation to the nucleus

**Figure 5:**
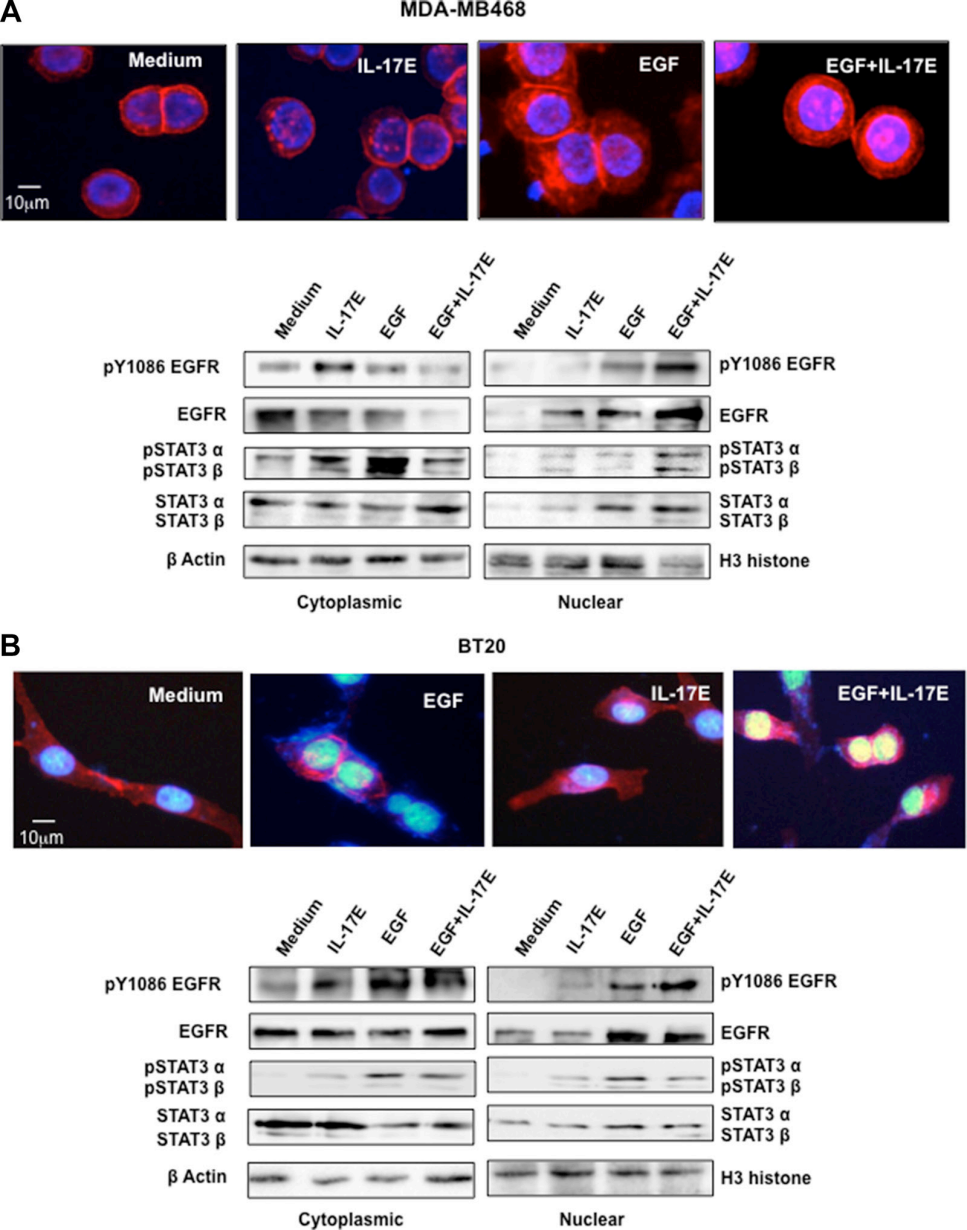
IL-17E facilitates pEGFR and pSTAT3 co-translocation to the nucleus MDA-MB468 (**A**) or BT20 (**B**) cells were stimulated with IL-17E (10 ng/ml), EGF (10 ng/ml) or a combination of both. In the upper panel of (A) and (B), EGFR localization, as assessed by immunostaining with anti-EGFR antibodies (red). Nuclei were visualized with DAPI (blue). In the lower panel of (A) and (B), translocation of EGFR, STAT3α/β, and their phosphorylated counterparts from the cytoplasm to the nucleus, as assessed by western blotting using specific antibodies. Anti-β actin and H3 histone antibodies were used as loading controls for the cytoplasmic and nuclear fractions, respectively. Data are representative of 2 independent experiments. Densitometric quantifications are presented in Supplementary Figure 1 (MDA-MB468) and Supplementary Figure 2 (BT20).

The translocation of phosphorylated EGFR to the nucleus is an integral component of the cascade that results in tumor resistance to EGFR therapeutic agents. Therefore, to determine the contribution of IL-17E to TNBC therapy resistance, we investigated the effect of IL-17E on EGFR nuclear translocation. Using immunofluorescence microscopy, we examined EGFR localization in TNBC cell lines stimulated with EGF, IL-17E, or both. In agreement with results from previous reports [21], EGF induced strong EGFR translocation from the membrane to the nucleus in MDA-MB468 TNBC cells (Figure 5A). Stimulation with IL-17E also induced EGFR translocation to the nucleus, but to a lesser extent than stimulation with EGF (Figure 5A upper panel). Importantly, the combination of IL-17E and EGF induced markedly increased EGFR translocation compared with that induced by each cytokine alone (Figure 5A upper panel).

To support these data, we subsequently isolated the cytoplasmic and nuclear fractions of IL-17E-, EGF- or IL-17E+EGF-stimulated MDA-MB468 cells and examined the levels of EGFR and its phosphorylated counterpart pY1086EGFR. Compared with EGF alone, IL-17E induced EGFR phosphorylation but did not induce significant EGFR nuclear translocation despite its capacity to induce non-phosphorylated EGFR nuclear translocation. Both forms of EGFR were translocated to the nucleus when MDA-MB468 cells were stimulated with the combination of IL-17E and EGF (Figure 5A lower panel and Supplementary Figure S1).

STAT3 binds the EGFR through a motif including pY1086 [22]. In addition, the correlation between pEGFR and pSTAT3α/β is well established and has been implicated in tumor resistance [23]. Therefore, we also examined STAT3 nuclear translocation and assessed the statuses of pSTAT3α and β, as well as those of their non-phosphorylated counterparts. IL-17E and EGF alone induced STAT3α and β translocation; however, the combination of the two agents triggered more significant pSTAT3α and β nuclear translocation than either agent alone.

To confirm these findings, we performed the above experiments with the BT20 cell line and obtained similar results. However, less EGFR and pSTAT3α and β translocation was induced by IL-17E and EGF in BT20 cells than in MDA-MB468 cells (Figure 5B and Supplementary Figure S2). This result is probably due to the inherent heterogeneity of TNBC tumor responses to various stimuli.

Together, these data indicate that the presence of IL-17E within the TNBC tumor microenvironment probably promotes and sustains EGFR activation and translocation and ultimately results in tumor resistance.

## DISCUSSION

Strategies for efficiently combating TNBC remain to be developed. This exploratory study assessed the IL-17E/IL-17RB pathway as a potential new target for the development of more efficient therapies for TNBC. Collectively, our data demonstrate that the IL-17E/IL-17RB pathway contributes to TNBC resistance to EGFR therapeutics through a loop that amplifies and sustains the phosphorylation of the main EGFR downstream kinases implicated in tumor resistance. Thus, blocking IL-17E or its receptor in conjunction with EGFR inhibitor administration may represent a novel strategy for treating these tumors.

We found that, consistently with the results of studies of hepatocyte growth factor (HGF) in breast cancer [11], Src and PYK2 activation also occurred downstream of the IL-17E receptor in TNBC cells. Inhibition of EGFR activation downstream of the IL-17E via administration of the Src-specific inhibitor AZM475271 supports this idea. IL-17E-induced Y1086EGFR phosphorylation in TNBC cells is dependent on EGFR kinase activity, as evidenced by the specific inhibition of this phosphorylation via administration of the EGFR kinase inhibitor Iressa. However, EGFR phosphorylation at Y1086 may also occur in the absence of EGFR kinase activity through pSrc/PYK2 crosstalk [21, 24]. The results obtained with IJG-1731 cells in this study support this idea and highlight the importance of pSrc/PYK2 crosstalk as a signaling checkpoint and molecular memory mechanism underlying tumor metastasis signaling [24].

The synergy between EGF and IL-17E is indicative of the pro-oncogenic role played by the IL-17 protein family in TNBC tumors. The presence of IL-17E in the TNBC tumor microenvironment may pre-activate the EGFR via Src/PYK2 crosstalk, thus resulting in enhanced sensitivity to EGF and potentially other EGFR ligands. Under these conditions, very low concentrations of EGFR ligands and weak expression (or accessibility) of this receptor are sufficient to activate tumor cells. Conversely, the above-mentioned increases in IL-17E-induced EGFR phosphorylation and nuclear translocation in the presence of EGF indicate that EGF also probably enhances the capacity of IL-17E to activate the EGFR and may serve as additional proof of the synergy between EGF and IL-17E with respect to sustaining EGFR activation in TNBC cells.

The EGFR is an important mediator of tumor development and progression, whereas IL-17E affects cell cycle progression, both in TNBC cells and in other breast cancer cells, such as human epidermal growth factor receptor 2 (HER2)-positive tumor cells [15]. Whether the effects of IL-17E on the cell cycle are dependent on the transactivation of EGFR or that of its family members (e.g., HER2) has not been determined. Nevertheless, the results described herein, together with those of our previous report, show the importance of IL-17A in pro-oncogenic signaling in breast cancer [25] and are indicative of the key roles played by IL-17 family members within the TNBC microenvironment. They also reinforce the idea that inflammation is a critical component of tumor progression [26, 27].

Our results demonstrate that IL-17E, in addition to its involvement in tumor progression [15], contributes to EGFR resistance. Our results provide the first evidence of IL-17E's involvement in EGFR remodeling and subcellular localization. Physiological EGF leads to EGFR degradation through receptor-mediated endocytosis and endosomal trafficking to lysosomes [28]. Therefore, IL-17E may alter EGFR degradation in malignant cells. Importantly, the nuclear fraction of EGFR contributes to Cetuximab resistance [10] and Iressa resistance [29]. IL-17E-mediated EGFR nuclear translocation, which is accompanied by pSTAT3 translocation, maintains EGFR phosphorylation and confers resistance to anti-EGFR therapeutic agents. Our data suggest that IL-17E-induced EGFR translocation facilitates the transport of pEGFR-associated pSTAT3 to the nucleus [22, 30]. In the nucleus, STAT3 activates the transcription of genes associated with tumor metastasis, as well as the transcription of anti-apoptotic and angiogenic genes, similarly to its function in various types of cancer [23, 31–33].

IL-17E-induced signaling is mediated through its specific heterodimer receptor IL17RA/IL17RB. Thus, IL-17E-induced resistance to anti-EGFR treatments conferred by Src activation and STAT3 and EGFR translocation is at least partially under the control of the IL-17E receptor, IL17RA/IL17RB, thus raising the question of whether it is involved in the treatment resistance associated with IL-17A and IL-17B, which share common co-receptors with IL-17E (IL-17RA and IL-17RB, respectively) [34]. Consistently with this idea, previous studies have shown that the IL-17RB/IL-17B signaling pathway promotes tumorigenicity and etoposide resistance in breast cancer cells through NFκB activation and Bcl-2 up-regulation [16]. Further investigation of these signaling mechanisms may improve the specificity and efficacy of biotherapies targeting these receptors.

Our findings advance the current understanding of anti-EGFR immunotherapy failures in breast cancer. IL-17E-induced signaling may also be interconnected with signaling mediated by other EGF receptor family members, such as HER2 and HER3, and contribute to their resistance to specific drugs. IL-17E is abundant in most metastatic tumors found in the brain [35, 36], liver [37] and lung [38]. However, whether IL-17E is pro-oncogenic or anti-oncogenic remains under debate.

Studies in animal models of colon cancer have demonstrated that IL-17E plays an inhibitory role with respect to the chronic inflammation associated with this disease [39]. Studies of hepatocellular carcinoma have shown that IL-17E activates the NFκB and Jak/STAT3 signaling pathways in cancer stem cells, thus resulting in tumor growth and progression and suggesting that IL-17E/IL-17RB may be a therapeutic target in the treatment of this disease [40]. In our TNBC model, direct IL-17E signaling via IL-17RB activated various signaling pathways associated with IL-17-induced tumor proliferation and progression [41, 42] and did not induce tumor cell apoptosis [15]. In contrast, Furata and colleagues have shown that IL-17E induces apoptosis in breast cancer cells [14], and Benatar and colleagues have reported that it exerts antitumor effects on xenografted tumors [43]. IL-17E is not the only member of IL-17 family that exerts contrasting effects in the setting of tumor progression. Direct or indirect Stat3 pathway activation by IL-17A promotes the proliferation and progression of various tumors, whereas IL-17A-mediated adaptive and innate immune responses exert anti-tumor effects [41, 42, 44–47]. The mechanisms underlying the contrasting roles played by members of the IL-17 family have not been fully elucidated. However, it is likely that IL-17 family members promote or suppress tumorigenesis in specific cell types and at specific stages of disease in response to specific cytokines present at the tumor site. The combination IL-17 signaling-mediated effects on tumor cell behavior at a specific stage of differentiation and various environmental factors probably determines whether tumor proliferation or apoptosis ultimately occurs.

In summary, our study provides the first evidence suggesting the possible role of IL-17E in tumor resistance to anti-EGFR therapeutic agents. Blocking either IL-17E or its receptor in combination with EGFR inhibitor administration might reduce the likelihood of tumor resistance and enhance therapeutic efficacy.

## MATERIALS AND METHODS

### Cell culture

BT20 and MDA-MB468 triple-negative (HER2-, ER-, PR-) cells were obtained from the American Type Culture Collection (ATCC N°HTB19 and ATCC N°HTB132, respectively). The LumB and Her2-, ER-, PR-negative IJG-1731 cell line was previously established in our laboratory, as described elsewhere [15], and was used as primary tumor cells model. Briefly, IJG-1731 were liberated from a patient LumB tumor biopsy characterized as an ypT2N1aM tumor, grown in culture media for several weeks for stabilization, and phenotyped as negative for estrogen, progesterone and HER2 receptors and positive for EGFR (HER1). BT20 and IJG-1731 cells were grown in complete RPMI-1640 medium with L-glutamine supplemented with 10% fetal calf serum (FCS) and penicillin–streptomycin solution (100 μg/ml each) (Life Technology, Saint-Aubain, France). MDA-MB468 cells were grown in a complete DMEM-F12 medium with glutamine, 10% FCS and penicillin–streptomycin. All cells were maintained in a humidified 5% CO_2_ atmosphere at 37°C. All experiments were conducted with confluent cells after overnight starvation.

### Antibodies and reagents

Rabbit anti-pEGFR (Y845), anti-pEGFR (Y1086), anti-pPYK2 (Y402), anti-pSTAT3 (Y705), anti-pSrc Family (Y416), anti-EGFR, anti-STAT3, anti-β-actin and Alexa 594-conjugated anti-rabbit F(ab)'2 fragment antibodies were purchased from Cell Signaling Technology (Danvers, MA, USA). Rabbit anti-Histone H3 antibodies were purchased from Thermo Scientific (Rockford, NY, USA). Isotype control IgG (MAB002) and anti-IL-17RB antibodies were purchased from R&D systems (Minneapolis, MN, USA). Iressa (Gefitinib) and the Src inhibitor AZM475271 were obtained from Tocris Bioscience (R&D systems). 7-AAD was purchased from Beckman (Coulter, France). EverBrite mounting medium with DAPI was purchased from Biotium (Hayward, CA, USA).

### Cell death

Cells (3 × 10^5^) were seeded in 6-well plates in complete medium for 24 hours and then starved overnight. Cells were stimulated for 48 hours at 37°C with IL-17E (10 ng/ml), Iressa (0.25, 0.5 or 1 μM) or a combination of both, as indicated. The cells were then harvested with cold PBS/0.5 mM EDTA, washed and stained with 7-AAD, according to the supplier's recommendations. Cell analysis was performed on a FC500 flow cytometer.

### Cellular fractionation

Cells (3 × 10^5^) were seeded in 6-well plates in complete medium for 24 hours and then starved overnight. The cells were stimulated for 2 hours at 37°C with IL-17E (10 ng/ml), EGF (10 ng/ml), or a combination of both, as indicated. The cells were then washed with PBS and lysed in lysis buffer (10 mM HEPES, 15 mM MgCl_2_, 10 mM KCl, 0.5 mM DTT, 0.2 mM PMSF, 1 mM Na_3_VO_4_, 10 mM NaF, and 0.5% Nonidet P-40). After the cells were incubated on ice for 20 minutes, the cytoplasmic fraction was obtained via centrifugation for 10 seconds at 10000 rpm at 4°C, and the nuclear pellet was washed with lysis buffer. For nuclear protein extraction, the isolated nuclei were suspended in buffer containing 20 mM HEPES, 25% glycerol, 420 mM NaCl, 15 mM MgCl_2_ and 0.2 mM EDTA supplemented with PMSF, DTT and Na_3_VO_4_, as above. After incubation for 20 minutes at 4°C, the nuclear extract was collected via centrifugation for 10 minutes at 10000 rpm. Protein concentrations were determined by using the Bradford method. Samples were mixed with Laemmli buffer, heated for 10 minutes at 95°C, and then subjected to 8% SDS-PAGE. Proteins were subsequently transferred to nitrocellulose membranes, hybridized with specific anti-EGFR, anti-pY1086EGFR, anti-STAT3 or anti-pY705STAT3 antibodies, and then detected with ECL. Anti-β-actin and anti-Histone H3 antibodies were used as loading controls.

### Tyrosine phosphorylation

Cells (3 × 10^5^) were seeded in 6-well plates in complete medium for 24 hours and then starved overnight. The cells were then stimulated with IL-17E (10 ng/ml), EGF (10 ng/ml), or a combination of both for 30 min in serum-free medium. The cells were then lysed in 1% Triton ×100 buffer and left on ice for 1 hour. Protein samples were then subjected to 8% SDS-PAGE. Western blotting was performed using specific antibodies to assess the phosphorylation of various kinases. In some experiments, cells were treated with AZM475271 (10 μM) or Iressa (0.25 μM) prior to stimulation with IL-17E, EGFR, or a combination of both, as indicated.

### Immunofluorescence microscopy

Cells (4.10^3^) were grown on Lab-Tek chamber slides in complete culture medium for 24 hours and then starved overnight. The cells were then stimulated with IL-17E (10 ng/ml), EGF (10 ng/ml) or a combination of both for 2 hours at 37°C, washed, and fixed with 4% paraformaldehyde (PFA)-PBS solution at 4°C. Cells were then permeabilized with 0.5% Triton-X-100 in PBS, saturated with 20% FCS in PBS and incubated 18 hours with anti-EGFR (1/500) in 10% FCS-PBS. Slides were mounted with mounting medium containing DAPI and then visualized with a Leica DMRB fluorescence microscope. Image analysis was performed with Archimed software (Microvision).

## SUPPLEMENTARY MATERIALS FIGURES



## References

[1] Marme F, Schneeweiss A Targeted Therapies in Triple-Negative Breast Cancer. Breast Care (Basel).

[2] Lehmann BD, Bauer JA, Chen X, Sanders ME, Chakravarthy AB, Shyr Y, Pietenpol JA Identification of human triple-negative breast cancer subtypes and preclinical models for selection of targeted therapies. J Clin Invest.

[3] Rakha EA, El-Sayed ME, Green AR, Lee AH, Robertson JF, Ellis IO (2007). Prognostic markers in triple-negative breast cancer. Cancer.

[4] Corkery B, Crown J, Clynes M, O'Donovan N (2009). Epidermal growth factor receptor as a potential therapeutic target in triple-negative breast cancer. Ann Oncol.

[5] Arteaga CL (2003). EGF receptor as a therapeutic target: patient selection and mechanisms of resistance to receptor-targeted drugs. J Clin Oncol.

[6] Bianco R, Troiani T, Tortora G, Ciardiello F (2005). Intrinsic and acquired resistance to EGFR inhibitors in human cancer therapy. Endocr Relat Cancer.

[7] Brand TM, Iida M, Dunn EF, Luthar N, Kostopoulos KT, Corrigan KL, Wleklinski MJ, Yang D, Wisinski KB, Salgia R, Wheeler DL Nuclear epidermal growth factor receptor is a functional molecular target in triple-negative breast cancer. Mol Cancer Ther.

[8] Han W, Lo HW Landscape of EGFR signaling network in human cancers: biology and therapeutic response in relation to receptor subcellular locations. Cancer Lett.

[9] Yarden Y, Pines G The ERBB network: at last, cancer therapy meets systems biology. Nat Rev Cancer.

[10] Li C, Iida M, Dunn EF, Ghia AJ, Wheeler DL (2009). Nuclear EGFR contributes to acquired resistance to cetuximab. Oncogene.

[11] Mueller KL, Hunter LA, Ethier SP, Boerner JL (2008). Met and c-Src cooperate to compensate for loss of epidermal growth factor receptor kinase activity in breast cancer cells. Cancer Res.

[12] Lee J, Ho WH, Maruoka M, Corpuz RT, Baldwin DT, Foster JS, Goddard AD, Yansura DG, Vandlen RL, Wood WI, Gurney AL (2001). IL-17E, a novel proinflammatory ligand for the IL-17 receptor homolog IL-17Rh1. J Biol Chem.

[13] Kim MR, Manoukian R, Yeh R, Silbiger SM, Danilenko DM, Scully S, Sun J, DeRose ML, Stolina M, Chang D, Van GY, Clarkin K, Nguyen HQ (2002). Transgenic overexpression of human IL-17E results in eosinophilia, B-lymphocyte hyperplasia, and altered antibody production. Blood.

[14] Furuta S, Jeng YM, Zhou L, Huang L, Kuhn I, Bissell MJ, Lee WH IL-25 causes apoptosis of IL-25R-expressing breast cancer cells without toxicity to nonmalignant cells. Sci Transl Med.

[15] Mombelli S, Cochaud S, Merrouche Y, Garbar C, Antonicelli F, Laprevotte E, Alberici G, Bonnefoy N, Eliaou JF, Bastid J, Bensussan A, Giustiniani J IL-17A and its homologs IL-25/IL-17E recruit the c-RAF/S6 kinase pathway and the generation of pro-oncogenic LMW-E in breast cancer cells. Sci Rep.

[16] Yin SY, Jian FY, Chen YH, Chien SC, Hsieh MC, Hsiao PW, Lee WH, Kuo YH, Yang NS Induction of IL-25 secretion from tumour-associated fibroblasts suppresses mammary tumour metastasis. Nat Commun.

[17] Huang CK, Yang CY, Jeng YM, Chen CL, Wu HH, Chang YC, Ma C, Kuo WH, Chang KJ, Shew JY, Lee WH Autocrine/paracrine mechanism of interleukin-17B receptor promotes breast tumorigenesis through NF-kappaB-mediated antiapoptotic pathway. Oncogene.

[18] Cooper JA, Howell B (1993). The when and how of Src regulation. Cell.

[19] Batzer AG, Rotin D, Urena JM, Skolnik EY, Schlessinger J (1994). Hierarchy of binding sites for Grb2 and Shc on the epidermal growth factor receptor. Mol Cell Biol.

[20] Park OK, Schaefer TS, Nathans D (1996). *In vitro* activation of Stat3 by epidermal growth factor receptor kinase. Proc Natl Acad Sci U S A.

[21] Verma N, Keinan O, Selitrennik M, Karn T, Filipits M, Lev S PYK2 sustains endosomal-derived receptor signalling and enhances epithelial-to-mesenchymal transition. Nat Commun.

[22] Shao H, Cheng HY, Cook RG, Tweardy DJ (2003). Identification and characterization of signal transducer and activator of transcription 3 recruitment sites within the epidermal growth factor receptor. Cancer Res.

[23] Bromberg JF, Wrzeszczynska MH, Devgan G, Zhao Y, Pestell RG, Albanese C, Darnell JE (1999). Stat3 as an oncogene. Cell.

[24] Park SY, Avraham HK, Avraham S (2004). RAFTK/Pyk2 activation is mediated by trans-acting autophosphorylation in a Src-independent manner. J Biol Chem.

[25] Cochaud S, Giustiniani J, Thomas C, Laprevotte E, Garbar C, Savoye AM, Cure H, Mascaux C, Alberici G, Bonnefoy N, Eliaou JF, Bensussan A, Bastid J IL-17A is produced by breast cancer TILs and promotes chemoresistance and proliferation through ERK1/2. Sci Rep.

[26] Fort MM, Cheung J, Yen D, Li J, Zurawski SM, Lo S, Menon S, Clifford T, Hunte B, Lesley R, Muchamuel T, Hurst SD, Zurawski G (2001). IL-25 induces IL-4, IL-5, and IL-13 and Th2-associated pathologies *in vivo*. Immunity.

[27] Wang YH, Angkasekwinai P, Lu N, Voo KS, Arima K, Hanabuchi S, Hippe A, Corrigan CJ, Dong C, Homey B, Yao Z, Ying S, Huston DP (2007). IL-25 augments type 2 immune responses by enhancing the expansion and functions of TSLP-DC-activated Th2 memory cells. J Exp Med.

[28] Wiley HS (2003). Trafficking of the ErbB receptors and its influence on signaling. Exp Cell Res.

[29] Huang WC, Chen YJ, Li LY, Wei YL, Hsu SC, Tsai SL, Chiu PC, Huang WP, Wang YN, Chen CH, Chang WC, Chen AJ, Tsai CH Nuclear translocation of epidermal growth factor receptor by Akt-dependent phosphorylation enhances breast cancer-resistant protein expression in gefitinib-resistant cells. J Biol Chem.

[30] Lin SY, Makino K, Xia W, Matin A, Wen Y, Kwong KY, Bourguignon L, Hung MC (2001). Nuclear localization of EGF receptor and its potential new role as a transcription factor. Nat Cell Biol.

[31] Niu G, Wright KL, Huang M, Song L, Haura E, Turkson J, Zhang S, Wang T, Sinibaldi D, Coppola D, Heller R, Ellis LM, Karras J (2002). Constitutive Stat3 activity up-regulates VEGF expression and tumor angiogenesis. Oncogene.

[32] Wei D, Le X, Zheng L, Wang L, Frey JA, Gao AC, Peng Z, Huang S, Xiong HQ, Abbruzzese JL, Xie K (2003). Stat3 activation regulates the expression of vascular endothelial growth factor and human pancreatic cancer angiogenesis and metastasis. Oncogene.

[33] Lo HW, Hsu SC, Ali-Seyed M, Gunduz M, Xia W, Wei Y, Bartholomeusz G, Shih JY, Hung MC (2005). Nuclear interaction of EGFR and STAT3 in the activation of the iNOS/NO pathway. Cancer Cell.

[34] Gaffen SL (2009). Structure and signalling in the IL-17 receptor family. Nat Rev Immunol.

[35] Pan G, French D, Mao W, Maruoka M, Risser P, Lee J, Foster J, Aggarwal S, Nicholes K, Guillet S, Schow P, Gurney AL (2001). Forced expression of murine IL-17E induces growth retardation, jaundice, a Th2-biased response, and multiorgan inflammation in mice. J Immunol.

[36] Sonobe Y, Takeuchi H, Kataoka K, Li H, Jin S, Mimuro M, Hashizume Y, Sano Y, Kanda T, Mizuno T, Suzumura A (2009). Interleukin-25 expressed by brain capillary endothelial cells maintains blood-brain barrier function in a protein kinase Cepsilon-dependent manner. J Biol Chem.

[37] Wang AJ, Yang Z, Grinchuk V, Smith A, Qin B, Lu N, Wang D, Wang H, Ramalingam TR, Wynn TA, Urban JF, Shea-Donohue T IL-25 or IL-17E Protects against High-Fat Diet-Induced Hepatic Steatosis in Mice Dependent upon IL-13 Activation of STAT6. J Immunol.

[38] Yao X, Sun Y, Wang W (2016). Interleukin (IL)-25: Pleiotropic roles in asthma. Respirology.

[39] Thelen TD, Green RM, Ziegler SF Acute blockade of IL-25 in a colitis associated colon cancer model leads to increased tumor burden. Sci Rep.

[40] Luo Y, Yang Z, Su L, Shan J, Xu H, Xu Y, Liu L, Zhu W, Chen X, Liu C, Chen J, Yao C, Cheng F Non-CSCs nourish CSCs through interleukin-17E-mediated activation of NF-kappaB, JAK/STAT3 signaling in human hepatocellular carcinoma. Cancer Lett.

[41] Sun Y, Pan J, Mao S, Jin J (2014). IL-17/miR-192/IL-17Rs regulatory feedback loop facilitates multiple myeloma progression. PLoS One.

[42] Wang L, Yi T, Kortylewski M, Pardoll DM, Zeng D, Yu H (2009). IL-17 can promote tumor growth through an IL-6-Stat3 signaling pathway. J Exp Med.

[43] Benatar T, Cao MY, Lee Y, Li H, Feng N, Gu X, Lee V, Jin H, Wang M, Der S, Lightfoot J, Wright JA, Young AH (2008). Virulizin induces production of IL-17E to enhance antitumor activity by recruitment of eosinophils into tumors. Cancer Immunol Immunother.

[44] Maniati E, Soper R, Hagemann T (2010). Up for Mischief? IL-17/Th17 in the tumour microenvironment. Oncogene.

[45] Yang B, Kang H, Fung A, Zhao H, Wang T, Ma D (2014). The role of interleukin 17 in tumour proliferation, angiogenesis, and metastasis. Mediators Inflamm.

[46] Qian X, Chen H, Wu X, Hu L, Huang Q, Jin Y (2015). Interleukin-17 acts as double-edged sword in anti-tumor immunity and tumorigenesis. Cytokine.

[47] Welte T, Zhang XH (2015). Interleukin-17 Could Promote Breast Cancer Progression at Several Stages of the Disease. Mediators Inflamm.

