# Supplementary material for: IL-17E synergizes with EGF and confers in vitro resistance to EGFR-targeted therapies in TNBC cells
Source: Oncotarget. 2016 Jul 23;7(33):53350–61. doi: 10.18632/oncotarget.10804 (PMC5288192; doi:10.18632/oncotarget.10804)
Supplement: Supplementary file 1 [file oncotarget-07-53350-s001.pdf]

IL-17E synergizes with EGF and confers *in vitro* resistance to EGFR-targeted therapies in TNBC cells

Supplementary Materials

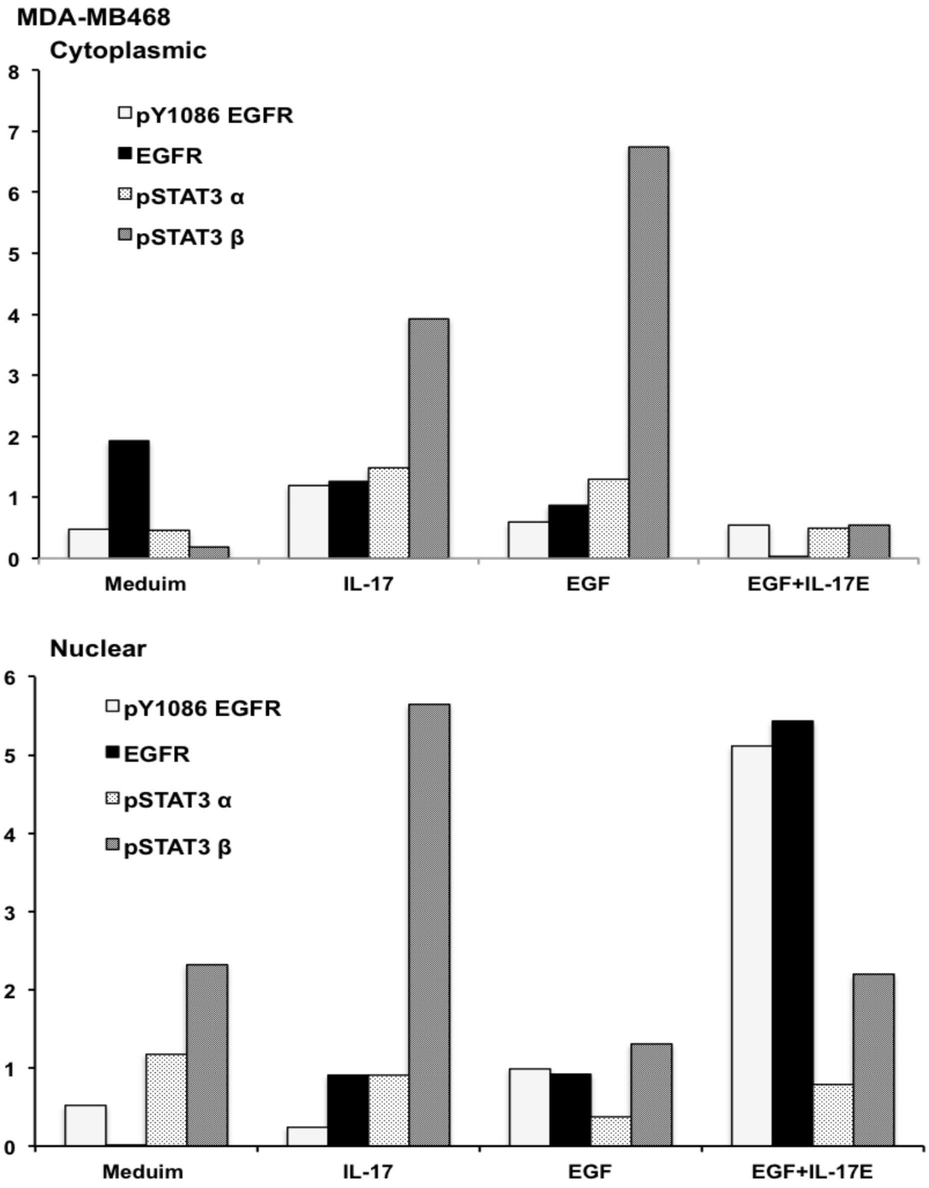

Supplementary Figure S1: IL-17E co-translocates pEGFR and pSTAT3 to the nucleus. MDA-MB468 cells were stimulated with IL-17E (10 ng/ml), EGF (10 ng/ml) or a combination of both. The translocation of EGFR, STAT3α/β, and their phosphorylated counterparts from the cytoplasm to the nucleus was assessed by Western blotting using specific antibodies. Densitometry quantification of pY1086 EGFR, EGFR, and pSTAT3α/β shown in the representative blots of Figure 5A is expressed as ratios between pY1086 EGFR, EGFR, and pSTAT3a/b and bactin (cytoplasmic) or H3 histone (nuclear).

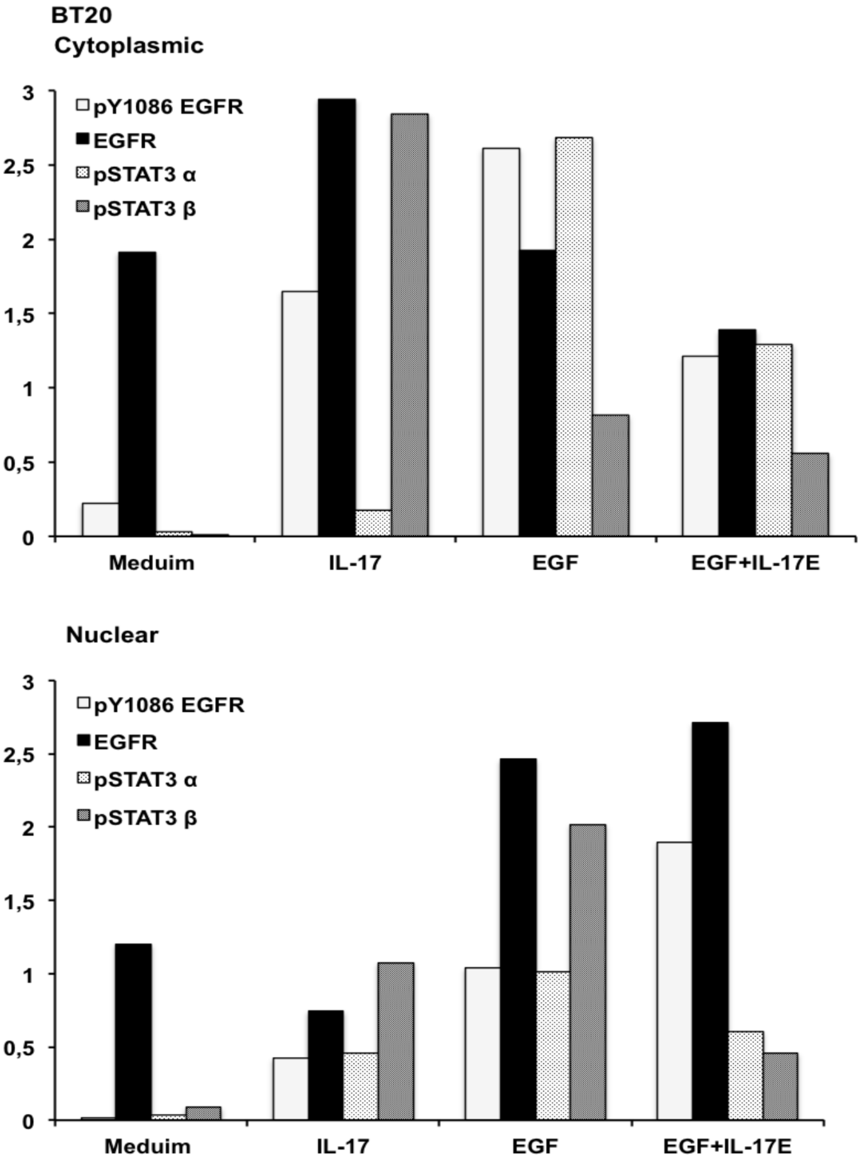

Supplementary Figure S2: IL-17E co-translocates pEGFR and pSTAT3 to the nucleus. BT20 cells were stimulated with IL-17E (10 ng/ml), EGF (10 ng/ml) or a combination of both. The translocation of EGFR, STAT3α/β, and their phosphorylated counterparts from the cytoplasm to the nucleus was assessed by Western blotting using specific antibodies. Densitometry quantification of pY1086 EGFR, EGFR, and pSTAT3α/β shown in the representative blots of Figure 5A is expressed as ratios between pY1086 EGFR, EGFR, and pSTAT3a/b and bactin (cytoplasmic) or H3 histone (nuclear).
